# How can we make international comparisons of infant mortality in high income countries based on aggregate data more relevant to policy?

**DOI:** 10.1186/s12884-017-1622-z

**Published:** 2017-12-19

**Authors:** Ania Zylbersztejn, Ruth Gilbert, Anders Hjern, Pia Hardelid

**Affiliations:** 1 The Farr Institute of Health Informatics Research, 222 Euston Road, London, NW1 2DA UK; 2 UCL Great Ormond Street Institute of Child Health, 30 Guilford Street, London, WC1N 1EH UK; 3 Centre for Health Equity Studies (CHESS), Stockholm University, SE–10691 Stockholm, Sweden; 4 Clinical Epidemiology Unit, Department of Medicine, Karolinska Institutet, 171 76 Solna, Stockholm Sweden

**Keywords:** Infant mortality, Neonatal mortality, Post-neonatal mortality, Stillbirth, International comparison, Birth weight

## Abstract

**Background:**

Infant mortality rates are commonly used to compare the health of populations. Observed differences are often attributed to variation in child health care quality. However, any differences are at least partly explained by variation in the prevalence of risk factors at birth, such as low birth weight. This distinction is important for designing interventions to reduce infant mortality. We suggest a simple method for decomposing inter-country differences in crude infant mortality rates into two metrics representing risk factors operating before and after birth.

**Methods:**

We used data from 7 European countries participating in the EURO-PERISTAT project in 2010. We calculated crude and birth weight-standardised stillbirth and infant mortality rates using Norway as the standard population. We decomposed between-country differences in crude stillbirth and infant mortality rates into the within-country difference in crude and birth weight-standardised stillbirth and infant mortality rates (metric 1), reflecting prenatal risk factors, and the between-country difference in birth weight-standardised stillbirth and infant mortality rates (metric 2), reflecting risk factors operating after birth. We also calculated birth weight-specific mortality.

**Results:**

Using our metrics, we showed that for England, Wales and Scotland risk factors before and after birth contributed equally to the differences in crude stillbirth and infant mortality rates relative to Norway. In Austria, Czech Republic and Switzerland the differences were driven primarily by metric 1, reflecting high rate of low birth weight. The highest values of metric 2 observed in Poland partially reflected high rates of congenital anomalies.

**Conclusions:**

Our suggested metrics can be used to guide policy decisions on preventing infant deaths through reducing risk factors at birth or improving the care of babies after birth. Aggregate data tabulated by birth weight/gestational age should be routinely collected and published in high-income countries where birth weight is reported on birth certificates.

**Electronic supplementary material:**

The online version of this article (10.1186/s12884-017-1622-z) contains supplementary material, which is available to authorized users.

## Background

Infant mortality is an important indicator of the health of a nation. It reflects the quality of obstetric and neonatal care, the health and welfare of women before conception and during pregnancy, and the health of children after birth [1–4]. Infant mortality is also strongly associated with public policies that impact on levels of poverty, income and employment support for parents [5].

International comparisons of infant mortality are a commonly used indicator for international organisations (for example the World Health Organisation (WHO) [6, 7], the Organisation for Economic Co-operation and Development (OECD) [3] and UNICEF [1]), policy makers and public health researchers as they demonstrate a country’s potential for preventing child deaths by reducing infant mortality rates to levels observed in countries with the lowest rates. For example, Scandinavian countries have some of the lowest infant mortality rates in the world and are commonly used for comparisons with the USA and UK, where infant mortality is among the highest of high-income countries [8–12].

Observed discrepancies in infant mortality can often be partly explained by variation in the prevalence of key risk factors at birth such as preterm birth (< 37 weeks), or low birth weight (< 2500 g) [2, 4, 13]. A key question for policy is when and how interventions should be targeted to prevent the largest number of deaths in early life: addressing maternal health and socio-economic circumstances before and during pregnancy, or improving the care of babies after birth, given their characteristics at birth? This distinction is often not obvious when comparing crude infant mortality rates, but could be determined using aggregate data tabulated by a key risk factor at birth (such as birth weight).

We demonstrate a simple method for international infant mortality comparisons. We describe two intuitive metrics which can inform policy makers about the extent to which any observed differences between countries relate to disparities in the prevalence of low birth weight (or prematurity), or to the risk of death in babies born with these risk factors. This decomposition of international differences in crude mortality rates can be used to guide policy decisions on preventing infant deaths through reducing risk factors at birth or improving the care of babies after birth.

## Methods

### Data

We used counts of live births, stillbirths, neonatal and post-neonatal deaths from perinatal health information systems collected by the EURO-PERISTAT project for 31 European countries. EURO-PERISTAT project aimed to collect comparable data about the health and care of mothers and babies in Europe to produce reliable and unbiased indicators of perinatal health [4]. Out of 31 participating countries, we included only countries that provided data tabulated by birth weight and gestational age for live births, stillbirths, neonatal and infant deaths. We further excluded regional data, countries with high proportion of missing data in any of the categories, countries with improbable counts of births and deaths per birth weight category (resulting from lack of linkage between birth and death registration data) or with other data quality issues, and countries with a small number of births per year, as their counts of deaths per birth weight category were prone to chance variations. Detailed exclusion criteria by country are presented in Additional file 1: Table S1. Seven remaining countries were included in the analyses. Some differences in registration practices remained (Additional file 1: Table S2), in particular, four countries included terminations of pregnancy in the stillbirth category [4].

In order to minimise bias due to inter-country differences in recording of live births and deaths at borderline viability and in legal limits for terminations of pregnancy (Additional file 1: Table S2), we excluded all births (live or still) weighing < 500 g [4, 14–16]. We also excluded births and deaths with missing birth weight (proportions of missing data are reported in Additional file 1: Table S3). We grouped birth weight as 500-999 g, 1000-1499 g, 1500-2499 g, ≥ 2500 g. Our analyses focused on birth weight, because methods used to calculate gestational age (e.g. ultrasound scan or last menstrual period) and their accuracy can vary between countries [17]. However, in the Additional file 1, we show that results were similar when we used gestational age (Additional file 1: Figures S1–S3 and Table S4).

### Statistical analysis

We calculated crude and birth weight-standardised mortality rates for each country. International comparisons of infant mortality are susceptible to bias due to inter-country differences in reporting of births and deaths that could lead to misclassification of stillbirths as early neonatal deaths (or vice versa) [16, 18, 19]. Therefore, we included counts of stillbirths in all analyses and we used the denominator of total births (including still- and live births) when calculating the rates [19]. We defined stillbirth and infant mortality rate as follows:

Equation 1 - Defining stillbirth and infant mortality rate and its subcomponents

$$ {\displaystyle \begin{array}{c} Stillbirth\& infant mortality rate=\frac{Infant Deaths and Stillbirths}{Total Births}\times 1000=\\ {}=\frac{Stillbirths}{Live Births+ Stillbirths}\times 1000+\frac{Neonatal Deaths}{Live Births+ Stillbirths}\times 1000\\ {}+\frac{Post- neonatal Deaths}{Live Births+ Stillbirths}\times 1000\end{array}} $$

We then needed to select a standard population. Usually this would be one with a low prevalence of low birth weight, since we are interested in calculating the maximum possible reduction in early life mortality attainable by preventing low birth weight births. This needs some consideration, since directly standardised mortality rates can vary depending on the choice of standard population. The difference in the crude mortality rate between country A and the standard population was then decomposed into two metrics as follows:

Equation 2 - Decomposition of the difference in crude mortality rates between country A and the standard population

$$ {\displaystyle \begin{array}{c} Crude mortality\ {rate}_{Country\ A}- Crude mortality\ {rate}_{Standard Population}=\\ {}= Crude mortality\ {rate}_{Country\ A}- Standardised mortality\ {rate}_{Standard Population}=\\ {}=\left( Crude mortality\ {rate}_{Country\ A}- Standardised mortality\ {rate}_{Country\ A}\right)\kern9.75em \left( metric\ 1\right)\\ {}+\left( Standardised mortality\ {rate}_{Country\ A}- Standardised mortality\ {rate}_{Standard Population}\right)\kern1.5em \left( metric\ 2\right)\end{array}} $$

Metric 1 is the within-country difference in the crude and birth weight-standardised mortality. It shows how the distribution of birth weight, particularly the prevalence of low birth weight, contributes to early life mortality in country A relative to the standard population. It indicates the number of stillbirths and infant deaths per 1000 births that could have been prevented if country A had the same proportion of low birth weight births as the standard population. Therefore, metric 1 is strongly associated with maternal health and socio-economic circumstances during pregnancy [4, 20, 21].

Metric 2 reflects the differences in birth weight-specific mortality between country A and the standard population. High values of metric 2 can be partly attributed to variation in the quality of care after birth, from the health service or at home, and partly to variation in the prevalence of other risk factors at birth, such as congenital malformations which are not adjusted for [21, 22]. We also demonstrate birth weight-specific mortality rates in each country to interpret high values of metric 2 and to identify characteristics of babies according to birth weight and age at death that might benefit most from strategies to reduce deaths.

## Results

**Fig. 1:**
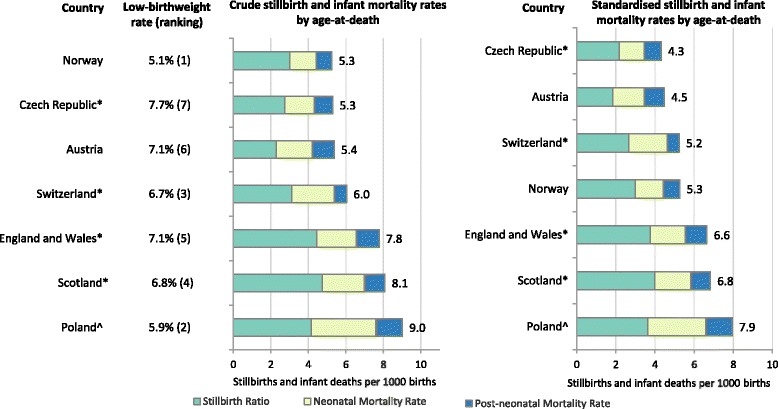
Rankings of countries based on crude and standardised stillbirth and infant mortality rates per 1000 births by age-at-death. The second column shows the proportion of births with low birth weight (< 2500 g). Countries with * included terminations of pregnancy in their counts of stillbirths; England, Wales and Scotland included terminations of pregnancy and stillbirths only after 24 weeks. ^In Poland access to terminations of pregnancy is restricted. All calculations were done given birth weight was non-missing and over 500 g

We demonstrate these metrics with Norway as the standard population, as Norway had the lowest proportion of low birth weight births (5.1%, Fig. 1). Norway also had the lowest crude stillbirth and infant mortality rates among the 7 countries in 2010 (5.3 stillbirths and infant deaths per 1000 births, Fig. 1, left-hand graph), and some of the lowest mortality rates for births weighing 500-999 g (Fig. 3, showing birth weight-specific mortality rates).

**Fig. 2:**
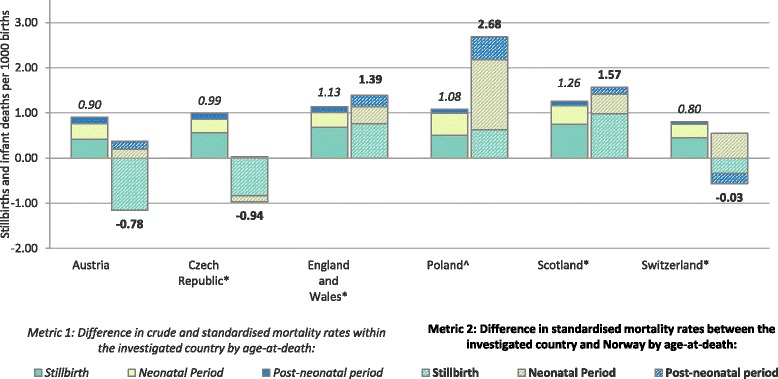
Decomposition of the difference in crude mortality rates per 1000 births between each country and Norway. Bars on the left-hand side represent metric 1; bars on the right-hand side represent metric 2. Countries with * included terminations of pregnancy in their counts of stillbirths; England, Wales and Scotland included terminations of pregnancy and stillbirths only after 24 weeks. ^In Poland access to terminations of pregnancy is restricted. All calculations were done given birth weight was non-missing and over 500 g

**Fig. 3:**
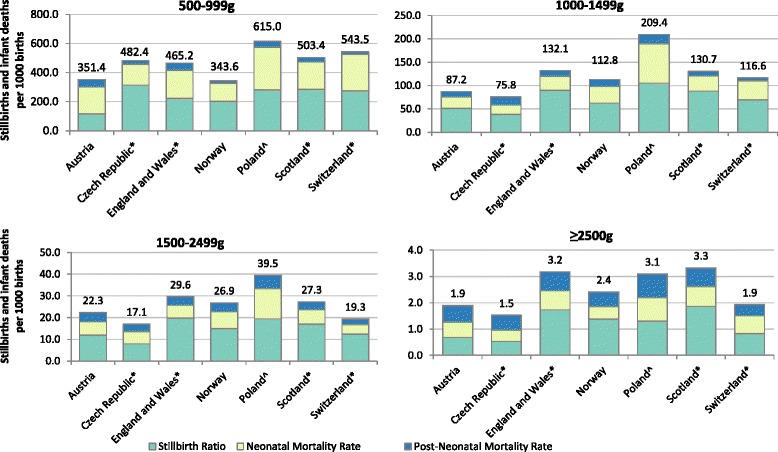
Birth weight-specific mortality rates per 1000 births in each country by age at death. Countries with * included terminations of pregnancy in their counts of stillbirths; England, Wales and Scotland included terminations of pregnancy and stillbirths only after 24 weeks. ^In Poland access to terminations of pregnancy is restricted. All calculations were done given birth weight was non-missing and over 500 g

Czech Republic and Austria had the lowest birth weight-standardised stillbirth and infant mortality rates (4.3/1000 and 4.5/1000 respectively, Fig. 1, right-hand graph). The two countries had some of the highest proportions of low birth weight births (7.1% in Austria and 7.7% in the Czech Republic, Fig. 1). The overall difference in crude stillbirth and infant mortality rates between these two countries and Norway was close to 0 (Fig. 1). However, the decomposition of the difference into the two metrics showed that approximately 1 stillbirth or infant death per 1000 births could have been prevented if Austria and the Czech Republic had the same birth weight distribution as in Norway (metric 1, Fig. 2). Negative values of metric 2 indicated lower birth weight-specific mortality rates than in Norway, especially for stillbirths and deaths in the neonatal period (Fig. 3). However, the rates for births weighing 500-999 g were higher than in Norway (Fig. 3).

The two metrics contributed almost equally to the difference in crude stillbirth and infant mortality rates between England, Wales and Scotland relative to Norway. If England and Wales reduced their low birth weight rate to that of Norway, 1.1 fewer stillbirth and infant deaths per 1000 births would have occurred in 2010 (metric 1, Fig. 2). A slightly higher reduction, 1.4/1000, could have been achieved if England and Wales had the same birth weight-specific mortality rates as in Norway (metric 2, Fig. 2). The differences in birth weight-specific mortality relative to Norway were largest in stillbirths weighing ≥ 1000 g, and neonatal and post-neonatal deaths in 500-999 g and ≥ 2500 g categories (Fig. 3). The results were similar in Scotland.

Poland had the highest crude and birth weight-standardised stillbirth and infant mortality rates (9.0/1000 and 7.9/1000 respectively, Fig. 1). The difference in crude stillbirth and infant mortality rates between Poland and Norway was driven primarily by the differences in birth weight-specific mortality – 2.7 stillbirths and infant deaths per 1000 births could have been prevented if Poland had the same birth weight-specific mortality as Norway (metric 2, Fig. 2). The differences were largest in the neonatal period for all birth weight categories and in the post-neonatal period for births weighing 500-999 g (Fig. 3). Approximately 1 death per 1000 births could have been prevented if Poland had the same distribution of birth weight as in Norway (metric 1, Fig. 2).

In Switzerland, the difference in crude mortality relative to Norway was primarily driven by unfavourable distribution of birth weight – 0.8 stillbirths or infant deaths per 1000 birth could have been prevented if Switzerland had the same prevalence of low birth weight as in Norway (metric 1, Fig. 2). Metric 2 was overall close to 0, but decomposition by age-at-death showed that Switzerland had higher birth weight-specific mortality in the neonatal period than Norway (metric 2, Fig. 2), especially in babies weighing 500-999 g and ≥ 2500 g (Fig. 3). Birth weight-specific mortality was lower, however, in the post neonatal period for all birth weight categories (Fig. 3).

## Discussion

The two presented metrics can provide policy makers with some insights into whether pre- or postnatal interventions are likely to have the largest effect on stillbirth and infant mortality rate. Most countries we examined would benefit from interventions reducing prevalence of low birth weight, reflected through metric 1. In England, Wales and Scotland the two metrics contributed almost equally to the difference in crude stillbirth and infant mortality rates relative to Norway. Large differences in neonatal and post-neonatal mortality for normal-birth weight babies could reflect socio-economic factors or high prevalence of congenital anomalies, while disparities in mortality among extremely-low birth weight babies could reflect differences in neonatal care for high-risk babies compared to Norway. For Austria, Czech Republic and Switzerland low birth weight was a major driver of differences in stillbirth and infant mortality rate relative to Norway. Czech Republic had some of the lowest stillbirth and neonatal mortality rates in almost all birth weight categories, reflecting good quality of neonatal and obstetric care. High stillbirth rate in 500-999 g category could be due to inclusion of terminations of pregnancy in the count of stillbirths. In Switzerland birth weight-specific mortality was lower than in Norway for all birth weight categories in the post-neonatal period, indicating good quality of infant care. The difference in crude stillbirth and infant mortality rates between Poland and Norway was driven primarily by the differences in birth weight-specific mortality (metric 2), especially in the neonatal period and for babies weighing < 1000 g in post-neonatal period, reflecting differences in obstetric and neonatal intensive care for high-risk babies. These differences can partly be explained by variation in prevalence of lethal congenital anomalies, since access to terminations of pregnancy is restricted in Poland.

Including stillbirths in the statistics helped to minimise impact of registration artefacts [19]. Results based on total births reflect the potential benefits from reducing potentially modifiable risk factors associated with both stillbirth and infant mortality (such as maternal obesity or smoking) [4, 23]. However, standardisation of the definitions is necessary. For example, in England, Wales and Scotland, the legal limit for registration of stillbirths was higher (≥ 24 weeks) than in other countries (≥ 22 weeks or ≥ 500 g), leading to possibly underestimated burden of stillbirths weighing 500 g-999 g [4]. In Czech Republic, England and Wales, Scotland and Switzerland, terminations of pregnancy (TOP) were included in stillbirth rates, but not in other countries [4]. The gestational age limit for TOP was < 24 weeks or lower (except for when mother’s life is in danger) in all countries apart from Switzerland. Excluding births at < 500 g helped to minimise the contribution of TOPs to stillbirth counts [24]. However, some TOPs could still be included in stillbirth counts for 500-999 g birth weight category in England & Wales, Scotland, Czech Republic and Switzerland. Separating data on stillbirths and terminations of pregnancy is needed for future comparisons [24]. Information about the timing of stillbirth (antepartum or intrapartum) could help distinguish between stillbirths due to prenatal risk factors or the quality of obstetric care. We were also not able to investigate the burden of high birth weight (≥ 4500 g) on infant mortality or conduct separate analyses for singleton and multiple births as such data was not reported in the EURO-PERISTAT project [4]. Furthermore, improvements in the quality of recorded data are needed – we were able to use only 7 out of 18 countries that reported all required data due to data quality issues such as missing data or lack of linkage between the registers, leading to implausible combinations of births and deaths per birth weight category.

Some of the results reflect variation in the prevalence of congenital anomalies, which we were not able to adjust for. Prevalence could vary due to differences in accessibility of antenatal screening, regulations regarding late terminations of pregnancy (as well as cultural differences affecting the uptake of terminations), and detection and recording of congenital anomalies. Repeating the analyses on counts excluding major congenital anomalies would help to account for these differences [2, 19].

Standardisation by birth weight or gestational age has been used in the past to quantify the contribution of low birth weight to international-differences in infant mortality [9, 12, 13]**.** It has been criticised, however, for introducing bias against populations with higher mean birth weight [25, 26]. Instead, some researchers focussed on birth weight-specific mortality [4, 22], although such comparisons do not account for differences in birth weight distributions and could be misleading [26]. Since both of these methods have limitations, we display both for a more informative comparison.

Our methods are simple and intuitive and can be easily applied to counts of births and deaths tabulated by age-at-death, birth weight and/or gestation. Such data needs to be routinely collected and reported by all countries to enable international comparisons of maternal and child health. The EURO-PERISTAT project has shown that many European countries (18 out of 31) already have the capacity to report such data if required, however improving the quality of collected data is still necessary [4]. More funding is needed, both in-country and for international collaborations such as EURO-PERISTAT, to ensure that such data is available in all countries and collected on a regular basis, using consistent definitions of stillbirths and live births, and ensuring complete recording of key risk factors including birth weight and gestational age for both live and stillbirth [15, 16, 23].

Further research is needed to assess which interventions would be most cost-effective at reducing pre- and post-natal risk factors within each country. A comparison of cause-specific mortality can identify modifiable factors operating after birth. For example, changes to infant sleeping position are associated with lower rates of sudden infant death syndrome (SIDS) [27]. Further, more detailed data on characteristics of babies and mothers are needed to identify modifiable risk factors operating before and during pregnancy. This could include information on risk factors such as maternal smoking during pregnancy and maternal body mass index (BMI). Thus, in countries where prevalence of smoking during pregnancy is high, smoking cessation programs could reduce the prevalence of low birth weight [28]. Therefore, in order to carry out detailed analyses of origins of inter-country disparities in infant mortality we need individual-level data with detailed information about characteristics of the baby and mother at birth and causes and timing of deaths from whole country birth cohorts based on administrative health and vital statistics databases. Such analyses require significant time investments to analyse, and the data are subject to access controls. In the meantime, careful use of aggregate data on all births and infant deaths tabulated by a key risk factor at birth offers the best available evidence to help policy makers develop preventive strategies to reduce stillbirth, neonatal and post-neonatal mortality.

## Conclusions

Careful use of tabulated aggregate data on all births and infant deaths tabulated by a risk factor at birth offers a quick yet simplified way to inform the design of preventive strategies to reduce stillbirth, neonatal and post-neonatal mortality. Our suggested metrics based on birth-weight-standardised and birth weight-specific stillbirth and infant mortality rates can provide some insights into whether pre- or postnatal interventions are likely to have the largest effect on stillbirth and infant mortality rates. Countries should routinely report counts of live births, stillbirths, neonatal and infant deaths tabulated by birth weight categories (and/or gestational age categories) to allow these metrics to be derived, however improvements to national registration systems and standardisation of definitions are needed to ensure comparability of the data.

## Additional file

Additional file 1: This document covers supporting materials for this study such as description of country exclusion criteria, description of registration practices in compared countries, additional analyses using data tabulated by birth weight, and sensitivity analyses based on data tabulated by gestational age. (DOCX 66 kb)

## Abbreviations

BMI: Body Mass Index
OECD: Organisation for economic co-operation and development
SIDS: Sudden infant death syndrome
TOP: Termination of pregnancy
WHO: World Health Organisation
