# Supplementary material for: How can we make international comparisons of infant mortality in high income countries based on aggregate data more relevant to policy?
Source: BMC Pregnancy Childbirth. 2017 Dec 19;17:430. doi: 10.1186/s12884-017-1622-z (PMC5738161; doi:10.1186/s12884-017-1622-z)
Supplement: Additional file 1: — This document covers supporting materials for this study such as description of country exclusion criteria, description of registration practices in compared countries, additional analyses using data tabulated by birth weight, and sensitivity analyses based on data tabulated by gestational age. (DOCX 66 kb) [file 12884_2017_1622_MOESM1_ESM.docx]

# Additional file 1

Table 1 – Details of country exclusion criteria for the study*

| **Country** | **Number of births per year** | **Detailed exclusion criteria** |
| --- | --- | --- |
| Austria | 78,989 | **Included in the study.** |
| Belgium | 133,508  (in 3 geographical regions) | Only regional coverage. |
| Cyprus | 8,602 | Did not provide all required data tabulated by birthweight and gestational age.  Small number of births per year. |
| Czech Republic | 116,920 | **Included in the study.** |
| Denmark | 63,513 | High rate of missing birth weight for stillbirths (29%). |
| England and Wales | 721,925 | **Included in the study.** |
| Estonia | 15,884 | Small number of births per year. |
| Finland | 61,421 | High rate of missing birth weight for stillbirths (22%). |
| France | 810 430 | Did not provide all required data tabulated by birthweight and gestational age. |
| Germany | 637,664 | Did not provide all required data tabulated by gestational age.  High rate of missing birth weight for infant deaths (22%). |
| Greece | 111,741 | Did not provide all required data tabulated by birthweight and gestational age. |
| Hungary | 90,920 | Recording error (more live births ≥2.5kg than total births ≥2.5kg), could be due to lack of linkage.  Did not provide all required data tabulated by gestational age. |
| Iceland | 4,903 | Recording error (total births at 500-999g < fetal loses and neonatal deaths at 500-999g), could be due to lack of linkage.  Small number of births per year. |
| Ireland | 75,595 | Did not provide all required data tabulated by birthweight and gestational age. |
| Italy | 547,569 | Did not provide all required data tabulated by birthweight and gestational age. |
| Latvia | 19,248 | Recording error (higher number of neonatal deaths at 500-999g than infant deaths fin that category), could be due to lack of linkage. |
| Lithuania | 30,977 | Did not provide all required data tabulated by birthweight and gestational age. |
| Luxembourg | 6,560 | High rate of missing birth weight and gestational age for infant deaths (38% respectively).  Small number of births per year. |
| Malta | 4,036 | Small number of births per year. |
| Netherlands | 178,838 | Did not provide all required data tabulated by birthweight and gestational age. |
| Northern Ireland | 25,692 | Recording error (the same number of stillbirth and neonatal deaths in all birthweight categories). |
| Norway | 62,612 | **Included in the study.** |
| Poland | 415,015 | **Included in the study.** |
| Portugal | 101,790 | Did not provide all required data tabulated by birthweight and gestational age. |
| Romania | 213,055 | High rate of missing gestational age for infant deaths (27%). |
| Scotland | 57,488 | **Included in the study.** |
| Slovakia | 55,825 | Did not provide all required data tabulated by birthweight and gestational age. |
| Slovenia | 22,416 | Recording error (higher number of neonatal deaths than infant deaths at 28-31 weeks), could be due to lack of linkage. |
| Spain | 400,415 | Did not provide all required data tabulated by birthweight and gestational age. |
| Sweden | 115,135 | Did not provide all required data tabulated by birthweight and gestational age. |
| Switzerland | 80,276 | **Included in the study.** |

Data comes from the Euro-Peristat project [1]

Table 2 – Differences in registration practices in included countries

| **Country** | **Definition of live birth** | **Definition of stillbirth** | **Terminations of pregnancy (TOP) included in stillbirth category** | **Gestational age limit for carrying out TOP (not including TOP when mother’s life is in danger)** |
| --- | --- | --- | --- | --- |
| **Austria** | ≥500g | ≥500g | NO | Late TOP rare |
| **Czech Republic** | ≥22 weeks | ≥22 weeks | YES | <24 weeks |
| **England and Wales** | ≥22 weeks | ≥24 weeks | YES | <24 weeks |
| **Norway** | ≥22 weeks | ≥22 weeks | NO | <22 weeks |
| **Poland** | ≥22 weeks | ≥500g | NO | Access to TOP restricted |
| **Scotland** | ≥22 weeks | ≥22 weeks; not complete at 22-23 weeks | YES | <24 weeks |
| **Switzerland** | ≥22 weeks, if missing ≥500g | ≥22 weeks, if missing ≥500g | YES | No limit |

Data comes from the Euro-Peristat project [1] and from Blonder et al. (the last column) [2].

Table 3 - Crude and standardised mortality rates per 1000 births with rankings and % of data with missing birthweight calculated for stillbirths and infant deaths by timing of death

| **Country** | **Austria** | **Czech Republic*** | **England and Wales*** | **Norway** | **Poland** | **Scotland*** | **Switzerland*** |
| --- | --- | --- | --- | --- | --- | --- | --- |
| Number of total births (with non-missing birthweight over 500g) | 78989 | 116912 | 721925 | 62632 | 415015 | 57488 | 80276 |
| **Crude Mortality Rates (per 1000 Births) for births with birthweight over 500g** | | | | | | | |
| Infant mortality and stillbirth rate (rank) | 5.38 (3) | 5.30 (2) | 7.78 (5) | 5.26 (1) | 9.02 (7) | 8.08 (6) | 6.04 (4) |
| Stillbirth ratio (rank) | 2.27 (1) | 2.73 (2) | 4.45 (6) | 3.01 (3) | 4.14 (5) | 4.74 (7) | 3.12 (4) |
| Neonatal rate (rank) | 1.94 (3) | 1.57 (2) | 2.12 (4) | 1.41 (1) | 3.43 (7) | 2.25 (5) | 2.26 (6) |
| Post-neonatal stay (rank) | 1.17 (5) | 1.00 (3) | 1.22 (6) | 0.85 (2) | 1.44 (7) | 1.10 (4) | 0.66 (1) |
| **Directly Standardised Mortality Rates (per 1000 Births) using Norway as the standard population, for births with birthweight over 500g** | | | | | | | |
| Infant mortality and stillbirth rate (rank) | 4.48 (2) | 4.32 (1) | 6.65 (5) | 5.26 (4) | 7.94 (7) | 6.83 (6) | 5.24 (3) |
| Stillbirth ratio (rank) | 1.85 (1) | 2.18 (2) | 3.77 (6) | 3.01 (4) | 3.64 (5) | 3.99 (7) | 2.67 (3) |
| Neonatal rate (rank) | 1.60 (3) | 1.27 (1) | 1.79 (4) | 1.41 (2) | 2.95 (7) | 1.84 (5) | 1.95 (6) |
| Post-neonatal stay (rank) | 1.03 (5) | 0.87 (3) | 1.10 (6) | 0.85 (2) | 1.35 (7) | 1.00 (4) | 0.61 (1) |
| **% of missing data per category** | | | | | | | |
| Live Births | 0.00% | 0.00% | 0.74% | 0.00% | 0.00% | 0.03% | 0.02% |
| Stillbirths | 0.00% | 0.00% | 5.16% | 13.76% | 0.00% | 4.23% | 0.79% |
| Neonatal Deaths | 0.00% | 2.14% | 9.18% | 0.00% | 0.00% | 6.52% | 0.00% |
| Post-neonatal deaths | 0.00% | 0.00% | 3.76% | 0.00% | 0.00% | 5.97% | 0.00% |

Countries with * included terminations of pregnancy in their counts of stillbirths. England and Wales and Scotland included terminations of pregnancy and stillbirths only after 24 weeks. In Poland access to terminations of pregnancy is restricted. Mortality rates were calculated given birth weight was non-missing and over 500g. % of missing data was calculated given birth weight was over 500g.

Figure 1 – Rankings of countries based on crude and standardised stillbirth and infant mortality rates per 1000 births by age at death.

The second column shows the proportion of preterm births (<37 weeks). Countries with * included terminations of pregnancy in their counts of stillbirths. England and Wales and Scotland included terminations of pregnancy and stillbirths only after 24 weeks. In Poland access to terminations of pregnancy is restricted. All calculations were done given gestational age was non-missing and over 24 weeks.

Figure 2 - Decomposition of the difference in crude mortality rates (per 1000 births) between each country and Norway.

Bars on the left-hand side represent metric 1; bars on the right-hand side represent metric 2. Countries with * included terminations of pregnancy in their counts of stillbirths. England and Wales and Scotland included terminations of pregnancy and stillbirths only after 24 weeks. In Poland access to terminations of pregnancy is restricted. All calculations were done given gestational age was non-missing and over 24 weeks.

Figure 3 – Gestation-specific mortality rates (per 1000 births) in each country by age at death.

Countries with * included terminations of pregnancy in their counts of stillbirths. England and Wales and Scotland included terminations of pregnancy and stillbirths only after 24 weeks. In Poland access to terminations of pregnancy is restricted. All calculations were done given gestational age was non-missing and over 24 weeks.

Table 4 - Crude and standardised mortality rates per 1000 births with rankings and % of data with missing gestational age calculated for stillbirths and infant deaths by timing of death. All calculations were done given gestational age was non-missing and over 24 weeks.

| **Country** | **Austria** | **Czech Republic*** | **England and Wales*** | **Norway** | **Poland** | **Scotland*** | **Switzerland*** |
| --- | --- | --- | --- | --- | --- | --- | --- |
| Number of total births (with non-missing gestational age over 24 weeks) | 78880 | 116638 | 714413 | 62292 | 414581 | 57393 | 80076 |
| **Crude mortality rates (per 1000 births) for births with gestational age <24 weeks** | | | | | | | |
| Infant mortality and stillbirth rate (rank) | 5.81 (4) | 4.88 (1) | 8.15 (6) | 5.46 (2) | 8.14 (5) | 8.28 (7) | 5.56 (3) |
| Stillbirth ratio (rank) | 2.99 (2) | 2.25 (1) | 4.97 (6) | 3.31 (4) | 3.64 (5) | 5.07 (7) | 3.02 (3) |
| Neonatal rate (rank) | 1.66 (3) | 1.63 (2) | 1.96 (5) | 1.28 (1) | 3.06 (7) | 2.09 (6) | 1.85 (4) |
| Post-neonatal stay (rank) | 1.15 (5) | 1.00 (3) | 1.22 (6) | 0.87 (2) | 1.44 (7) | 1.12 (4) | 0.69 (1) |
| **Directly standardised mortality rates (per 1000 births) using Norway as the standard population, for births with gestational age <24 weeks** | | | | | | | |
| Infant mortality and stillbirth rate (rank) | 4.93 (2) | 4.44 (1) | 7.12 (6) | 5.46 (4) | 7.85 (7) | 7.11 (5) | 5.21 (3) |
| Stillbirth ratio (rank) | 2.55 (2) | 2.02 (1) | 4.32 (7) | 3.31 (4) | 3.53 (5) | 4.31 (6) | 2.83 (3) |
| Neonatal rate (rank) | 1.35 (2) | 1.47 (3) | 1.68 (4) | 1.28 (1) | 2.91 (7) | 1.75 (6) | 1.70 (5) |
| Post-neonatal stay (rank) | 1.03 (4) | 0.95 (3) | 1.13 (6) | 0.87 (2) | 1.41 (7) | 1.05 (5) | 0.67 (1) |
| **% of missing data per category** | | | | | | | |
| Infant mortality and stillbirth rate | 0.00% | 0.00% | 0.96% | 0.90% | 0.00% | 0.04% | 0.05% |
| Stillbirth ratio | 0.00% | 0.00% | 2.95% | 2.37% | 0.00% | 0.00% | 0.00% |
| Neonatal rate | 0.00% | 3.06% | 2.72% | 0.00% | 0.00% | 2.44% | 0.00% |
| Post-neonatal stay | 0.00% | 0.00% | 1.13% | 5.26% | 0.00% | 4.48% | 0.00% |

Countries with * included terminations of pregnancy in their counts of stillbirths. England and Wales and Scotland included terminations of pregnancy and stillbirths only after 24 weeks. In Poland access to terminations of pregnancy is restricted Mortality rates were calculated given gestational age was non-missing and over 24 weeks. % of missing data was calculated given gestational age was over 24 weeks.

References

1. Euro-Peristat project with SCPE and Eurocat: ***European Perinatal Health Report. The health and care of pregnant women and babies in Europe in 2010*.** May 2013, **:**.

2. Blondel B, Cuttini M, Hindori-Mohangoo AD, Gissler M, Loghi M, Prunet C, Heino A, Smith L, van der Pal-de Bruin K, Macfarlane A, Zeitlin J, Euro-Peristat Scientific Committee: **How do late terminations of pregnancy affect comparisons of stillbirth rates in Europe? Analyses of aggregated routine data from the Euro-Peristat Project.** BJOG 2017, .
